# Exploring the Experiences of Persons Living With Dementia and Their Care Partners During the COVID-19 Pandemic

**DOI:** 10.1093/geroni/igab046.2005

**Published:** 2021-12-17

**Authors:** Jeanine Yonashiro-Cho, Elizabeth Avent, Laura Mosqueda, Zachary Gassoumis

**Affiliations:** 1 University of Southern California, Alhambra, California, United States; 2 University of Southern California, Los Angeles, California, United States; 3 Keck School of Medicine of USC, Alhambra, California, United States

## Abstract

Public health measures implemented to mitigate the spread of COVID-19 have transformed the physical and social environments in which we live. The effects of these policies on persons living with dementia (PLWD) and their care partners (CPs) are not fully understood. This study explores the experiences, attitudes, and perceptions of caregiving dyads during the COVID-19 pandemic. Cross-sectional survey data were drawn from a larger longitudinal study examining the relationship between PLWD aged 65+ and their CPs being conducted in a metropolitan city significantly affected by COVID-19. Interviews with were conducted remotely via videoconferencing and telephone. Data on sources and types of care provided for the PLWD, relationship quality and conflict, and caregiver stress were collected and analyzed using descriptive statistics and tests of independence. Preliminary results from PLWD (n=8) and CPs (n=13) confirmed a reduction in social interaction with family members and friends. CPs reported they (n=5) or other family members (n=2) changed their schedules to provide care for the PLWD. CPs reported increased conflict with the PLWD regarding care provision, going out or welcoming visitors, and home management. In contrast, PLWD reported a lack of conflict among household members (n=6) and the perception of good changes (n=2) and increased quality time with CPs. Preliminary findings provide empirical evidence of the effects of pandemic public health policies on dyads enrolled in this study and reveal differences in perceived relational conflict between PLWD and their CPs. Further research is needed to better understand the experiences of dyads and develop supportive interventions.

